# Anxiety and Depression in Patients With Pulmonary Arterial Hypertension in Northwest China: A Cross-Sectional Study

**DOI:** 10.3389/fpsyt.2021.758120

**Published:** 2022-02-04

**Authors:** Juxia Zhang, Yuhuan Yin, Yujie Wen, Fugui Shi, Jiancheng Wang

**Affiliations:** ^1^Clinical Educational Department, Gansu Provincial Hospital, Lanzhou, China; ^2^School of Nursing, Gansu University of Chinese Medicine, Lanzhou, China; ^3^Cardiovascular Department, Gansu Provincial Hospital, Lanzhou, China; ^4^Lanzhou Hand and Foot Surgery Hospital, Lanzhou, China; ^5^Geriatrics Department, Gansu Provincial Hospital, Lanzhou, China

**Keywords:** depression disorder, anxiety disorder, pulmonary arterial hypertension, patients, China

## Abstract

**Objective:**

Pulmonary arterial hypertension (PAH) is a rare life-threatening and incurable disease. Although symptoms of depression and anxiety have been widely reported, these traits and associated factors have not been systematically assessed in Northwest China.

**Methods:**

A cross-sectional study was conducted between March 2020 and February 2021. 106 PAH patients in Northwest China were evaluated by Self-rating Anxiety Scale (SAS) and the Self Rating Depression Scale (SDS) questionnaire.

**Results:**

Overall, the included patients had particularly high depressive symptoms (70.09%), while anxiety among them was 17.55%. Multivariate linear regression revealed that patients with lower age (*p* = 0.04), female (*p* **<** 0.01), smoking (*p* **<** 0.01), WHO functional class III/IV (*p* **<** 0.01), higher mean pulmonary hypertension (*p* **<** 0.01), lower left ventricular ejection fraction (*p* **<** 0.01), and lower 6-min walking distance (*p* **<** 0.01) had higher anxiety scores. Patients who lived in rural areas (*p* = 0.01), smoking (*p* **<** 0.01), WHO functional class III/IV (*p* **<** 0.01), higher mean pulmonary hypertension (*p* = 0.04), lower 6-min walking distance (*p* **<** 0.01), and college degree or above had higher depression scores (*p* = 0.02).

**Conclusions:**

Mental health problems such as depression are common among patients with PAH in Northwest China. Patients' characteristics such as smoking status, WHO functional class, and 6-min walking distance were related to anxiety and depression scores. Thus, early detection of mental health problems such as depression and anxiety should be detected in PAH patients. Meanwhile, interventions against these problems should be used to improve such patients' mental status.

## Introduction

Pulmonary hypertension (PH) is defined as mean pulmonary artery pressure (PAP) at rest of >25 mmHg (1). It is divided into five major categories (2). Currently, one that is of particular clinical relevance is pulmonary arterial hypertension (PAH) (1). An internationally registered epidemiological data on PAH showed that the prevalence of PAH was 15 cases per million adults (2), making it a rare disease. However, even with PAH-specific drug treatment, there is yet no cure for it and survival for PAH patients remains poor (3). Koudstaal et al.'s study showed that among newly diagnosed PAH cases, the 5-year survival rate was 61.2% (4).

PAH patients can experience multiple symptoms, including shortness of breath (5), fatigue, chest discomfort, and decreased physical function (6). These changes can significantly affect psychological and physical conditions of patients in different ways. For example, PAH patients may have feelings of uncertainty about their future, significant economic burden (7), a high risk of maternal mortality (30–55%) (7, 8), and heavy side effects of medical therapies (9). All of these could have a negative effect on a patient's mental status, resulting in the occurrence of depression and anxiety symptoms. To better understand the health of PAH patients, mental status (depression and anxiety) is increasingly considered as an important indicator of an individual's psychological health (7). In other countries, PAH patients showed a high mental health burden. For example, a study in Japan reported 64% of candidates had depression and 28% had anxiety (10). A study in Beijing, China, found 66.3% of samples had depression and anxiety (11), while 38.2% of PAH patients reported these symptoms in Germany (12). Some studies also have suggested that patients' anxiety and depression symptoms may further lead to deterioration of physical function (11, 13), poor cardiac function (14), poor prognosis (11), worsening quality of life, and increased health-related costs (10).

The present study was produced in Gansu Provincial Hospital. The tertiary hospital is located in Lanzhou, the capital of Gansu Province, which is located in Northwest China. Affected by geographical and regional conditions, compared with other provinces in China, the development of economy, culture, and information as well as medical resources supplying Gansu has been relatively less. According to China's comprehensive economic competitiveness development report, Gansu was listed as 27th among 31 provinces (15). The average altitude of Gansu Province is 2,158 m (3,370.6–421.9 m). At present, there are no detailed epidemiological data of PAH in Gansu. In 2017, Gansu Provincial Hospital took the lead in setting up an outpatient clinic for pulmonary hypertension in the province, and carried out a voluntary activity to care for “Blue Lips,” so that this group of people receive attention. In 2020, about 300 inpatients with pulmonary hypertension have been diagnosed and treated in the hospital. With the standardization of disease diagnosis and treatment, the number of patients with pulmonary hypertension is increasing. However, these patients are mostly treated by cardiologists who had less experience in detecting the patients' mental status (16). Guidelines for the diagnosis and treatment of pulmonary hypertension in China (2021 edition) suggest the standard management for PAH patients (17), and the psychological aspects of the disease are often neglected due to the lack of structured psychosomatic support, which also showed in other countries (16). Many mental health epidemiological studies in patients with PAH have been conducted. However, only a few surveys exist in China. With the increase of patients diagnosed with PAH, in addition to clinical treatment, we should investigate the following: (1) How many patients with PAH have symptoms of depression and anxiety? (2) Is there any association between patients' characteristics and incidence of depressive and anxiety, so as to provide effective interventions and lead to improved comprehensive healthcare for PAH patients?

## Methods

### Study Design

This is a cross-sectional study that uses a questionnaire to evaluate symptoms of depression and anxiety in selected PAH patients.

### Setting and Participants

The study included hospitalized patients diagnosed with PAH at Gansu Provincial Hospital from March 2020 to February 2021.

For inclusion, patients should meet the following criteria: (1) diagnosed with PAH; (2) aged 18 years or older; (3) under optimized medical therapy for PAH for at least 2 months. The diagnosis of PAH was established according to the current guidelines (18). Exclusion criteria were: (1) impaired cognition and judgment; (2) history of diagnosed psychological problems (such as depression and anxiety)—this information was obtained through asking for medical history of mental disorders; (3) unable to communicate; (4) severe comorbidity (such as untreated left heart disease). The nature of the study was explained to all samples, and subsequently, all of them gave verbal consent.

### Questionnaire

A questionnaire was formed to collect information from samples. The first section was demographic data of selected patients, which include age, gender, marital status, education level, home location, smoking, drinking, WHO functional class, mean arterial pressure (mean PAP), left ventricular ejection fraction (LVEF%) and 6-min walking distance (6MWD). The second part consisted of 20 items from Self-rating Anxiety Scale (SAS) to assess symptom of anxiety current or in the last week (19). Each item was answered with “a little of the time,” “some of the time,” “good part of the time,” or “most of the time.” For scoring of the answer, items 5, 9, 13, 17, and 19 were positive rated on a 4–1 scale whereas others were negative rated on a 1–4 scale. Based on standardized scoring algorithm, symptom of anxiety was defined if the SAS score ≥50 points (50–59 mild, 60–69 moderate, ≥70 severe). The third part included 20 items from the Self Rating Depression Scale (SDS) that used to measure symptoms of depressive using a 4-point scale “none” for 1, “a little of the time” for 2, “most of the time” for 3, and “all of the time” for 4 (20). Under standardized scoring algorithm, depression symptom was defined if the SAS score was ≥53 points (53–62 mild, 63–72 moderate, >72 severe). The higher scores indicate more severe symptoms.

### Sample Size

According to a preliminary survey, the prevalence of depression in patients with pulmonary hypertension was 40.2%. We assume *p* = 50% and a precision level of 10% (50 ± 10%). The sample size was calculated as follows (19):

(1) $$\begin{array}{r} \text{n}=\frac{Z_{\alpha /2}^{2}\left(1-p\right)p}{\delta^{2}} \end{array}$$

where confidence level *Z*_(α/2)_ = 1.96 and δ is the allowable error (0.10). The resulting sample size of 96 was increased to 10–12% to account for questionnaires discarded due to lack of information and filling errors.

### Data Collection

Two trained researchers performed the survey. Patients' demographic and clinical symptom data including gender, age, WHO functional class, 6MWD, mean PAP, and main symptoms were traced from medical records. Then, the researchers invited individual patients to a single room next to the cardiovascular medicine department. One of the researchers explained the nature of the study and verbal agreement was acquired from patients. Each respondent was given 20 min to fill in the questionnaire. If someone cannot correctly answer for some reasons (such as illiteracy), one of the family members would be invited to assist. After all patients completed the survey, the two researchers entered the data into Excel 2007.

### Statistical Methods

Data were imported from Excel to SPSS 21 for analyses. Categorical variables were expressed in absolute (*n*) and relative (%) frequencies, and numerical variables were expressed in mean ($\bar{x}$) and SD. *T*-test and one-way ANOVA were performed to preliminarily analyze various independent variables related to anxiety and depression scores. Next, multiple linear analysis was used to examine the independent factors related to anxiety and depression scores. The dependent variable was anxiety or depression scores, taking into account confounding factors; all demographic and disease-related characteristics were included in the regression model. During multivariable modeling, tolerance and the variance inflation factor (VIF) were used to detect multicollinearity. Any predictor with a tolerance below 0.1 and/or a VIF above 10 was excluded from the final model. A *p*-value < 0.05 was considered to be statistically significant.

## Results

### Basic Information of the Participants

As shown in Table 1, of 129 included patients, 20 were excluded as WHO functional class or 6MWD was not performed. Furthermore, three patients were excluded for not completing the SAS. Finally, 106 patients were enrolled (consent rate = 82.17%) with mean age 54.74 ± 14.43 (range: 23–85). Of them, 54.72% were female. According to WHO functional classifications (WHO FC), the majority (62.26%) of patients were classified as WHO FC III/IV. The mean PAP was 43.62 ± 13.24 (range: 26–73). Regarding common symptoms, 75.47% reported shortness of breath, followed by cough (35.85%) and chest pain (13.21%).

**Table 1:** Participant demographics.

**Characteristics**		**Participants** **(*N* = 106)**
		***n* (%)**
Age (years), $\bar{x}$ *± SD*, range		54.74 ± 14.43, 23–85
Gender	Male	48 (45.28)
	Female	58 (54.72)
Marital status	Unmarried	10 (9.43)
	Married	96 (90.57)
Home location	Rural	48 (45.28)
	Urban	58 (54.72)
Educational level	High school or below	74 (69.81)
	College degree or above	32 (30.19)
Profession	Worker or farmers	60 (56.60)
	Cadre or retired	46 (43.40)
Smoking	Yes	18 (16.98)
Drinking	Yes	14 (13.21)
WHO-FC, *n* (%)	Class I/II	40 (37.74)
	Class III/IV	66 (62.26)
Mean PAP (mmHg), $\bar{x}$ ± SD, range		43.62 ± 13.24, 26–73
LVEF%, $\bar{x}$ ± SD, range		51.51 ± 8.07, 32–69
6MWD (m), $\bar{x}$ ± SD, range		354.03 ± 80.47, 136–489
Common symptom	Shortness of breath	80 (75.47)
	Chest pain	14 (13.21)
	Cough	38 (35.85)
Antidepressant drug (yes)		0 (0.00)
Oxygen therapy (yes)		106 (100)

*PAP, pulmonary arterial pressure; LVEF%, left ventricular ejection fraction; 6MWD, 6-min walking distance; WHO-FC, World Health Organization functional class*.

### Symptoms of Depression and Anxiety

**Figure 1:**
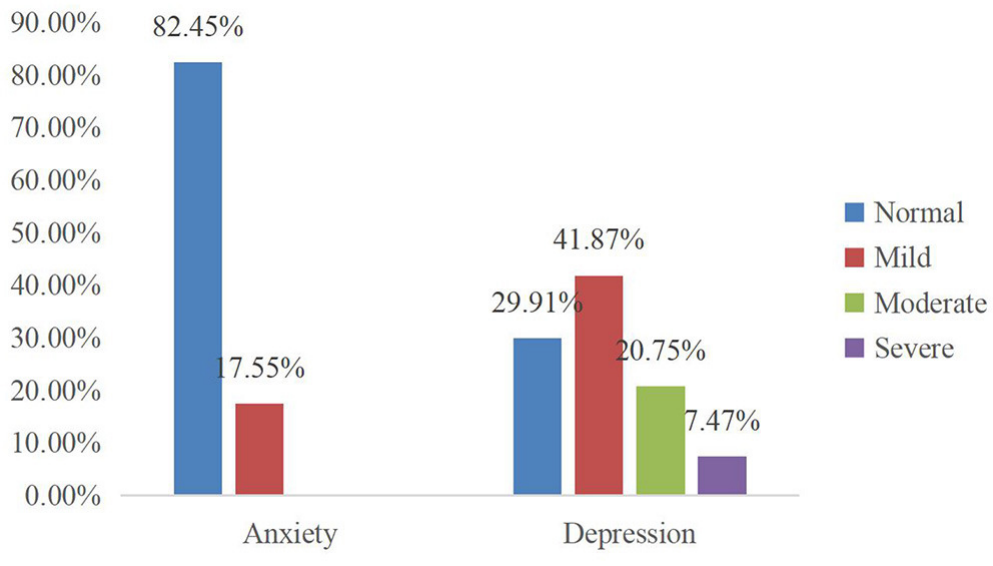
The percentage of anxiety and depression levels.

Of the 106 participants, 70.09% had symptoms of depression with 28.22% moderate to severe (Figure 1); however, no one used antidepressant drugs or accepted non-drug interventions (Table 1).

The incidence of depressive symptoms in urban PAH patients was significantly higher than those in rural areas (*t* = 3.42, *p* **<** 0.01). Non-smokers had significantly higher depression scores than smokers (*t* = 3.29, *p* **<** 0.01), while the depression scores were higher among those with LVEF% <50 (*t* = 2.60, *p* = 0.01) and higher mean PAP (Table 2). Anxiety symptoms were found by 17.55% of the participants, and they were all mild anxiety, while none were found to have moderate or greater anxiety (Figure 1). The incidence of anxiety was significantly higher among PAH patients whose homes were located in an urban area (*t* = 2.13, *p* = 0.03). Anxiety symptoms were significantly higher among participants who were not smoking (*t* = 5.11, *p* **<** 0.01) and not drinking (*t* = 2.46, *p* = 0.01) compared with those who did (Table 2).

**Table 2:** Univariate analysis of anxiety, depression scores, and general characteristics.

**Characteristics**		**Anxiety score**			**Depression score**		
		**$\bar{\text{x}}$ ± SD**	***F/t***	***p***	**$\bar{\text{x}}$ ± SD**	***F/t***	***p***
Age	<40	38.89 ± 6.25	1.45^a^	0.23	45.28 ± 3.31	1.02^a^	0.36
	40 ~ 60	39.88 ± 3.72			48.50 ± 7.05		
	>60	41.68 ± 5.05			47.40 ± 6.56		
Gender	Male	39.84 ± 5.41	−0.89^b^	0.37	46.46 ± 6.86	−1.17^b^	0.24
	Female	41.90 ± 4.34			48.28 ± 5.86		
Marital status	Single	37.25 ± 5.75	1.75^b^	0.08	45.25 ± 2.61	1.82^b^	0.08
	Married	40.50 ± 4.70			47.69 ± 6.59		
Home location	Rural	39.06 ± 5.20	2.13^b^	**0.03**	44.69 ± 6.78	3.42^b^	**<0.01**
	Urban	41.55 ± 4.38			49.74 ± 5.36		
Educational level	High school or below	40.44 ± 4.87	0.72^b^	0.48	48.69 ± 6.81	2.02^b^	0.13
	College degree or above	41.53 ± 4.54			50.84 ± 5.59		
Profession	Worker or farmers	38.55 ± 4.35	2.92^b^	0.09	46.35 ± 7.850	1.03^b^	0.36
	Cadre or retired	42.40 ± 3.65			47.89 ± 4.352		
Smoking	No	41.65 ± 4.46	5.11^b^	**<0.01**	48.55 ± 5.488	3.29^b^	**<0.01**
	Yes	34.45 ± 3.79			42.09 ± 8.444		
Drinking	No	40.98 ± 4.86	2.46^b^	**0.01**	47.91 ± 5.757	1.52^b^	0.13
	Yes	36.79 ± 3.80			44.46 ± 9.296		
WHO-FC	Class I/II	39.78 ± 4.41	1.42^b^	0.15	47.63 ± 8.044	0.15^b^	0.87
	Class III/IV	41.50 ± 5.41			48.35 ± 5.125		
Mean PAP (mmHg)	26 35	41.86 ± 5.69	2.14^a^	0.12	46.64 ± 7.694	7.65^a^	**<0.01**
	36 45	41.96 ± 3.50			46.94 ± 4.794		
	>45	42.15 ± 4.71			48.40 ± 6.085		
LVEF%	<50	43.63 ± 5.08	−0.11^b^	0.90	50.06 ± 5.067	2.60^b^	**0.01**
	50 70	38.64 ± 4.11			45.99 ± 6.717		
6MWD (m)	>426	38.58 ± 6.80	1.24^a^	0.29	40.89 ± 9.414	1.03^a^	0.36
	150 425	40.49 ± 4.52			47.99 ± 5.488		
	<150	42.50 ± 4.16			52.25 ± 4.185		

a *Equal to F value*.

b *Equal to t value*.

*WHO-FC, World Health Organization functional class; PAP, pulmonary arterial pressure; LVEF%, left ventricular ejection fraction; 6MWD, 6-min walking distance*.

*Bold values are those indicating statistical significance*.

### Influencing Factors for Symptoms of Anxiety and Depression

The results of multivariate linear regression analysis (Table 3) showed that the significant influencing factors of anxiety scores were age (95% CI, −0.12, −0.002; *p* = 0.04), gender (95% CI, −4.59, −0.92; *p* **<** 0.01), smoking (95% CI, −10.18, −4.17; *p* **<** 0.01), WHO-FC (95% CI, 1.13, 2.81; *p* **<** 0.01), mean PAP (95% CI, 1.07, 3.19; *p* **<** 0.01), LVEF% (95% CI, −0.39, −0.16; *p* **<** 0.01), and 6MWD (95% CI, −0.05, −0.008; *p* **<** 0.01) (Table 3).

**Table 3:** Multiple linear regression analysis of anxiety scores and general characteristics.

**Characteristics**	**Unstandardized** **coefficients**		**Standardized coefficients**	**t**	***p***	**95% CI**	
	**B**	**SE**	**Beta**			**Lower**	**Upper**
Age	−0.06	0.03	−0.19	−2.06	**0.04**	−0.12	−0.002
Gender (male)	−2.75	0.95	−0.28	−2.98	**<0.01**	−4.59	−0.92
Marital status (married)	0.94	1.79	0.05	0.52	0.60	−2.62	4.51
Home location (rural)	−1.64	0.94	−0.17	−1.74	0.08	−3.52	0.22
Educational level (college degree or above)	0.21	0.31	0.05	0.68	0.49	−0.40	0.82
Profession (cadre or retired)	0.08	0.26	0.03	0.31	0.75	−0.44	0.61
Smoking (yes)	−7.17	1.51	−0.55	−4.74	**<0.01**	−10.18	−4.17
Drinking (yes)	2.15	1.46	0.15	1.47	0.14	−0.75	5.05
WHO-FC (class III/IV)	1.97	0.42	0.36	4.66	**<0.01**	1.13	2.81
Mean PAP	2.13	0.53	0.39	4.03	**<0.01**	1.07	3.19
LVEF%	−0.27	0.05	−046	−4.72	**<0.01**	−0.39	−0.16
6MWD	−0.02	0.01	−0.35	−2.73	**<0.01**	−0.05	−0.008

*Adjusted R^2^ = 0.536, p < 0.01*.

*Bold values are those indicating statistical significance*.

*95% CI, 95% confidence interval for B*.

The significant influencing factors of depression scores were home location (95% CI, −5.91, −0.81; *p* = 0.01), smoking (95% CI, −10.27, −2.09; *p* **<** 0.01), WHO functional class (95% CI, 1.36, 3.48; *p* **<** 0.01), mean PAP (95% CI, 0.46, 3.92; *p* = 0.04), 6MWD (95% CI, −0.09, −0.03; *p* **<** 0.01), and educational level (95% CI, 0.10, 1.61; *p* = 0.02) (Table 4).

**Table 4:** Multiple linear regression analysis of depression scores and general characteristics.

**Characteristics**	**Unstandardized** **coefficients**		**Standardized** **coefficients**	**t**	***p***	**95% CI**	
	**B**	**SE**	**Beta**			**Lower**	**Upper**
Age	−0.03	0.04	−0.07	−0.79	0.42	−0.11	0.05
Gender (male)	−1.99	1.26	−0.15	−1.58	0.11	−4.49	0.50
Marital status (married)	−1.02	2.44	−0.04	−0.41	0.67	−5.88	3.84
Home location (rural)	−3.36	1.28	−0.26	−2.61	**0.01**	−5.91	−0.81
Educational level (college degree or above)	0.85	0.38	0.27	2.25	**0.02**	0.10	1.61
Profession (cadre or retired)	0.36	0.36	0.11	1.00	0.31	−0.35	1.08
Smoking (yes)	−6.18	2.06	−0.36	−3.00	**<0.01**	−10.27	−2.09
Drinking (yes)	1.98	1.98	0.10	1.00	0.32	−1.96	5.94
WHO functional class (class III/IV)	2.42	0.53	0.45	4.58	**<0.01**	1.36	3.48
Mean PAP	2.19	0.86	0.31	2.55	**0.04**	0.46	3.92
LVEF%	−0.14	0.08	−0.18	−1.85	0.06	−0.30	0.01
6MWD	−0.06	0.01	−0.62	−4.65	**<0.01**	−0.09	−0.03

*Adjusted R^2^ = 0.498, p < 0.01*.

*Bold values are those indicating statistical significance*.

*95% CI, 95% confidence interval for B*.

## Discussion

This study is the first to provide evidence regarding prevalence of psychiatric disorders in patients with PAH in Northwest China, thus providing essential references for the psychological treatment of PAH patients.

Our study suggested that the prevalence of depression was considerably high in patients with PAH. Notably, we found that the state of depression and anxiety was significantly related to social characteristics including smoking status, WHO functional class, mean PAP, and 6MWD in these patients.

### Anxiety and Depression Symptoms of Patients With PAH

A previous study estimated that the incidence of moderate to severe depression in PAH patients has been between 20 and 50% (21). Our study also found similar results: 70.09% of patients were judged to have depressive symptoms on the depression score, and 28.22% had moderate to severe depression. The result of the high incidence of depressive symptoms in present study was consistent with a previous study in Beijing, China (11), and in other countries (12). These may be because of the poor prognosis, significant side effects, and activity limitations of patients diagnosed of PAH that may expose patients to higher stressors, leading to the development of depression and anxiety (21). High levels of such status can adversely affect health outcomes (22), increase the chance of unhealthy behaviors (23), cause adverse events to occur more frequently (11), and reduce quality of life (10). However, there was a lack of psychological support for PAH patients with depression in the present population, who have not received any type of intervention, and similar observations have been made in the study of Harzheim et al. (24). It is noteworthy that these patients in our hospital are mostly treated by pulmonologists or cardiologists with limited experience in the detection of mental illness, and similar limited sources of medical care have been observed in other developing countries (22). The lack of mental health detection for PAH patients will lead to inadequate diagnosis, treatment, and intervention for these problems. Since this negative mental status will bring adverse outcomes to PAH patients, and psychological support has not been the standardized management in most pulmonary hypertension institutions (22), as a high-risk group for developing emotional problems in the literature, we suggest that early screening and diagnosis of mental disorders in this group is essential. Meanwhile, to improve mental symptoms of patients with PAH, the need for a psychological support and counseling would be beneficial (25).

In contrast, the anxiety was less prevalent than depression in the present study with prevalence of 17.55%, which was similar to the finding from Japan (10). One study found that patients who had long been diagnosed and followed in a reference center have a lower frequency of psychological disorders than those who had recently been diagnosed with PAH (25). Although a small proportion of patients experienced anxiety in the present study, our study was a survey at a given point of time and did not compare mental health change with the disease progression. In addition, the present study only focused on depression and anxiety symptoms, it will be necessary to analyze the association between mental status and quality of life in the future.

### Effect of Social Characteristics on Symptoms of Depression and Anxiety

Our study observed that PAH patients from urban areas were more likely to have psychological problems than those from rural areas. Similar outcomes were also found in residents of China (26–28) and other countries (29). A meta-analysis concluded that the odds of depression increased by about 30% in cities compared with rural areas (30). Even though urban living can offer benefits [such as convenient transportation, access to medical resources (28)], it is characterized by a lack of green space, intensive social activity, intense competition, and social fragmentation (lack of social connections between individuals in a particular geographical area) (31, 32), which may have a negative impact on those susceptible to mental illness (29). Thus, appropriate interventions are needed for PAH patients with mental disorders in different geographic regions. Our study found that patients who smoked had lower scores for depression and anxiety compared with those who did not. This is probably because nicotine, the main ingredient in cigarettes, binds to nicotinic acetylcholine receptors, which increases the amount of dopamine secreted by neurons in the brain's reward centers, leading to feelings of happiness and relaxation (33). Besides, some studies have also found that withdrawal from cigarettes after nicotine addiction can lead to increased anxiety and depression symptoms (34, 35).

Our multiple linear regression showed that the likelihood of depression and anxiety in patients with better functional status (WHO FC I or II) was significantly lower. FC and 6MWD are two measures to assess patients' functional exercise capacity and treatment efficacy (36), which are also specific treatment goals for PAH patients as guidelines recommend with FC I–II and 6MWT ≥380–440 m (37). One study has shown that the prevalence of depression increased with functional class (FC) worsening (38) and 6MWT decreasing (22). Exercise and physical activity are useful to gain self-confidence (39), which are also good opportunities to meet or socialize with others and can help improve one's mood (40). However, severe physical capacity impairment was common in patients with PAH (21). In the present study, most individuals had FC III/IV, and the average of 6MWD was 354.03 ± 80.47 m, which was similar to the results in America (41), Brazil (42), and France (43). For many individuals diagnosed with PAH, impaired exercise tolerance is a prominent feature that can lead to increased sense of social isolation (22). The occurrence of mental disorders is obviously related to the restriction of daily activities (11). However, it is still difficult to determine whether exercise restriction is the cause of mental illness. It has been reported that proper exercise training is an adjunct to improving the exercise capacity of PAH patients (11); in addition, patients who were followed at PAH centers were less likely to develop mental disorders than those who were not (11), further demonstrating the positive role of psychological support. Diagnosed patients are prone to emotional problems due to the type and severity of PAH; in the future, there should be more evidence on the role of interventions, including psychological counseling and appropriate social support for PAH patients with anxiety and depression.

There are limitations in this study. First, this was a cross-sectional study and did not prove a causal relationship between the prevalence of mental disorders and social characteristics. Second, the samples were chosen from a tertiary hospital in Northwest China, and the use of a single site may result in sampling bias that affects the representativeness of the study. Third, our study focused on inpatients who may have higher rates of depression and anxiety than outpatients. Finally, the use of self-reported patient data may lead to some bias because the answers may be exaggerated or underreported.

## Conclusions

The study concluded that although optimized treatments for PAH were available, these patients often experience symptoms of depression and anxiety, which were associated with impaired cardiac function and mobility. Also, these problems were underestimated due to a lack of standardization in the psychological detection. The study calls for screening and diagnosis of anxiety and depression to be included in routine clinical testing. In addition, more randomized controlled trials should be conducted to explore the effects of interventions such as psychological counseling, psychosocial support, and drugs on PAH patients with anxiety and depression.

## Data Availability Statement

The raw data supporting the conclusions of this article will be made available by the authors, without undue reservation.

## Ethics Statement

The studies involving human participants were reviewed and approved by Research Committee of Gansu Provincial Hospital. Written informed consent for participation was not required for this study in accordance with the national legislation and the institutional requirements. Written informed consent was not obtained from the individual(s) for the publication of any potentially identifiable images or data included in this article.

## Author Contributions

JZ was responsible for study design, implementation of the study, and drafting of the article. JW and YY were responsible for study design, statistical analysis, data interpretation, and drafting of the article. YW was responsible for data collection and data interpretation. YY and FS were responsible for implementation of the study and data interpretation. YY revised the contents of the article. All authors contributed to the article and approved the submitted version.

## Funding

This research was funded by the Natural Science Foundation of Gansu Province (Grant Nos. 21JR7RA613 and 21JR7RA607).

## Conflict of Interest

The authors declare that the research was conducted in the absence of any commercial or financial relationships that could be construed as a potential conflict of interest. The reviewer BM declared a shared affiliation, with no collaboration, with one of the author FS to the handling editor at the time of the review.

## Publisher's Note

All claims expressed in this article are solely those of the authors and do not necessarily represent those of their affiliated organizations, or those of the publisher, the editors and the reviewers. Any product that may be evaluated in this article, or claim that may be made by its manufacturer, is not guaranteed or endorsed by the publisher.
